# Calcium response via CRAC channels in human synovial cells induced by shear stress in rheumatoid arthritis

**DOI:** 10.1016/j.jphyss.2025.100013

**Published:** 2025-03-06

**Authors:** Yu Okumura, Kanya Honoki, Yasuhito Tanaka, Miyako Takaki, Keiji Asada

**Affiliations:** a Department of Orthopaedic Surgery, Nara Medical University School of Medicine, Kashihara, Nara, Japan; b Department of Physical Therapy, Faculty of Health Science, Osaka University of Human Science, Osaka, Japan; c Department of Rehabilitation, Faculty of Health Science, Suzuka University of Medical Science, Mie, Japan

**Keywords:** Rheumatoid arthritis, Fibroblast-like synovial cells, Shear stress, Calcium release-activated calcium channel

## Abstract

The role of calcium release-activated calcium channel (CRAC) inhibitors in the pathogenesis of rheumatoid arthritis (RA) is unclear. We focused on stromal interaction molecule 1 (STIM1) and Ca^2+^ release-activated channel regulator 2 A (CRACR2A), which participate in CRAC activation, to understand the signaling mechanism of human RA fibroblast-like synovial (FLS) cells in response to shear stress (SS). Human normal and RA FLS cell cultures were studied. The rates of intracellular calcium release and extracellular calcium influx in response to SS differed, and the responses to the first and second stimuli were analyzed. In the RA FLS cells, CRAC inhibitor significantly decreased the second/first stimulus ratio compared with that of the normal cells, and STIM1 and CRACR2A exhibited significantly increased expression levels compared with those in the normal FLS cells. Therefore, STIM1 and CRACR2A expression and Ca^2+^ influx in FLS cells are implicated in the pathogenesis of RA.

## Highlights

• CRAC participates in the pathogenesis of RA.
• CRAC activation is higher in RA FLS cells than in normal FLS cells.
• Intracellular Ca^2+^ concentration regulation in RA FLS cells is CRAC-dependent.
• TRPV4 influences intracellular Ca^2+^ level regulation independent of the RA status.

## Introduction

Rheumatoid arthritis (RA) is a chronic inflammatory and systemic autoimmune disease characterized by the inflammation of the synovial membrane [1], [2]. As the disease progresses, synovial cells proliferate and produce cytokines, such as interleukin-1 beta (IL-1β), interleukin-6 (IL-6), and granulocyte-macrophage colony-stimulating factor [3], [4], [5], and express the receptor activator of nuclear factor-kappa B ligand (RANKL) [6]. These factors cause the worsening of joint physiology.

RA is an autoimmune disease, and normal mechanical stimuli, such as exercise, are believed to induce the release of inflammatory cytokines and the progression of inflammation. The advent of disease-modifying antirheumatic drugs (DMARDs) [7], which prevent the progression of joint destruction, has increased the number of patients with RA who maintain their mobility and daily living abilities. However, there are no established strategies to protect fragile joints from daily activity stress and maintain joint function in patients with RA. When cells are subjected to mechanical stress, mechanosensitive ion channels undergo structural changes in response to pressure and shear stress (SS), altering ion permeability and increasing intracellular calcium concentration [8], [9], [10].

A decreased calcium ion concentration in the endoplasmic reticulum (ER) activates the calcium release-activated calcium channel (CRAC), the store-operated Ca^2+^ entry (SOCE). CRAC inhibitors reportedly exert protective effects against bone and cartilage destruction during the pathogenesis of RA [11]. In a study, enhanced CRAC expression and function were observed in the peripheral T cells of patients with active RA, and CRAC gene silencing restored the abnormal function of the peripheral T cells [12]. In addition, systemic CRAC gene silencing via lentivirus reduced inflammation and autoimmune responses in a collagen-induced arthritis model [13]. Aberrant SOCEs disrupt Ca^2+^-sensitive signaling pathways, causing abnormal gene expression, cell differentiation, cytokine secretion, and migration [14], [15]. Therefore, CRAC is being investigated as a target for the development of new immunosuppressive agents [16], [17].

Upon CRAC activation, calcium release-activated calcium modulator 1 (Orai1), stromal interaction molecule 1 (STIM1), and Ca^2+^ release-activated channel regulator 2 A (CRACR2A) bind and transport calcium ions into the cell. CRACR2A is a Ca^2+^ sensor in the cytoplasm and regulates CRAC channel activity by modulating the formation of the STIM1-Orai1 complex. CRAC regulates intracellular Ca^2+^ influx and opens in response to ER Ca^2+^ depletion, triggering signaling cascades that induce T cell activation and inflammatory responses characterized by cytokine production [18]. Thus, we hypothesized that CRACR2A regulates the intracellular calcium dynamics induced by mechanical stimulation. However, the precise role of CRAC in the pathogenesis of RA remains unclear. Evaluating the involvement of CRAC in intracellular calcium-mediated signaling induced by mechanical stress in synovial cells would facilitate the development of novel therapies for RA. In this study, we aimed to elucidate the signaling mechanism in human RA synovial cells by applying mechanical stress to the joints.

## Materials and methods

### Cell culture

Human normal and RA fibroblast-like synovial (FLS) cells (CDD-H-2910-N, CDD-H-2910-RA; Articular Engineering, IL, US) were cultured in matrigel (356230, Corning Inc, USA) -coated 35-mm diameter glass bottom dishes (P35–0-14-C, MatTek Corporation, USA) for 11 days in a Dulbecco’s modified Eagle’s medium (DMEM) (041–29775, Fujifilm-Wako Pure Medical Corporation, Japan) supplemented with fetal bovine serum (SFBM30–0500, Equitech-Bio, inc, USA) and penicillin-streptomycin (15070063, Thermo Fisher Scientific, USA). All cultures were maintained at 37 °C in a 5 % CO_2_ atmosphere. Third-passage cells were used in this experiment.

### SS

Puff applications with nitrogen gas were ejected from a glass micropipette (PINS30 (20)−00FT; Prime Tech, Ibaraki, Japan) with outer and inner diameters of approximately 30 and 20 µm, respectively. This application was validated in a previous study [19]; however, a fluid flow was used rather than a nitrogen gas. A pneumatic picopump (PV830, World Precision Instruments, FL, USA) was used for nitrogen gas ejection at an intra-micropipette pressure of 48.3 kPa for 10 ms. The micropipette was attached to a micromanipulator (MHW-3; Narishige, Tokyo, Japan) at 45° to the dish bottom plane and set to 50 µm above the perinuclear region of the target FLS cell in the center of the microscopic field. Puff stimulation has been used as a mechanical stimulus [20], [21], [22] and as a method of mechanical stimulation to the transient receptor potential ion channel family [23], [24].

### Ca^2+^ imaging

Before measurement, the cells cultured for 11 days were incubated for 60 min at 37 °C in a culture medium containing fluo 3-AM (2 μM; DOJINDO, Kumamoto, Japan) and washed twice with a HEPES buffer (140 mM NaCl, 5 mM KCl, 0.6 mM MgCl_2_, 1 mM CaCl_2_, 10 mM HEPES, 1 mg/mL D-glucose, 1 mg/mL BSA, and 0.2 mg/mL sulfinpyrazone; adjusted to pH 7.4).

The [Ca^2+^]_i_ changes were monitored using a digital imaging system (AQUACOSMOS, Shizuoka, Japan) mounted on an inverted microscope (IX-73; Olympus, Tokyo, Japan). Fluo 3-containing cells were excited at 488 nm, and the intensity of the fluorescent emission from the indicator was recorded at 515–565 nm.

Digital Ca^2+^ images were normally collected at 157 ms intervals, and the fluorescence intensity at a given time (F_t_) was normalized to that at the start (F_0_), yielding relative values representing the integrated [Ca^2+^]_i_. A positive Ca^2+^ fluorescence response was defined as an increase in intracellular fluorescence levels > 10 % of that of the baseline (F_t_/F_0_ >1.1). In these experiments, a custom-made dish chamber was used for local perfusion. The chamber containing cells was superfused with a HEPES buffer at 2 mL/min and maintained at 35 °C during Ca^2+^ imaging.

### Experimental groups

The experimental samples were divided into the control (vehicle, a HEPES buffer), CRAC blockade (1 μM CRAC inhibitor [YM-58483; R&D Systems, MN, USA]), and transient receptor potential vanilloid 4 (TRPV4) channel blockade (1 μM TRPV4 antagonist [HC-067047; Sigma Aldrich, MO, USA]) groups. When CRAC is activated by Ca^2+^ depletion in the ER, Orai1, STIM1, and CRACR2A bind and transport Ca^2+^ into the cell; however, CRAC inhibitors act on Orai1 to prevent Ca^2+^ uptake.

Superfusion with a HEPES buffer containing a blocker or inhibitor was performed 15 min before the first SS. The second to the first responses SS were compared, as previously reported [25], [26].

### Immunocytochemical analysis

For immunocytochemical analysis, the cells were rinsed twice with phosphate-buffered saline (PBS), fixed for 15 min with 4 % paraformaldehyde diluted in PBS, washed thrice for 5 min in PBS, and permeabilized with 0.1 % v/v Triton X-100. After fixation, the cultures were washed twice with PBS and blocked with 0.2 % bovine serum in PBS for 30 min. Primary antibodies were diluted (1:250 for rabbit polyclonal anti-CRACR2A antibody [66787–1-IG, Proteintech, IL, USA] and 1:250 for rabbit polyclonal anti-TRPV4 antibody [ab191580, Alomone Labs, Jerusalem, Israel]) and incubated for 1 h at 37.5 °C. CRACR2A immunoreactivity was determined using an Alexa Fluor 488-conjugated secondary antibody (Alexa Fluor 488 goat anti-rabbit; Molecular Probes; 1:500 in PBS) for 2 h in the dark at 25 °C. TRPV4 immunoreactivity was assessed using an Alexa Fluor 594-conjugated secondary antibody (Alexa Fluor 594 donkey anti-rabbit; Molecular Probes, 1:500). The tissues were examined using a confocal microscope (FV1000; Olympus). Final images were constructed using FV10-ASW [Ver1.7] Olympus). The fluorescence intensity of the images was quantitatively analyzed using the ImageJ software (NIH, Bethesda, MD, USA). Fluorescence values of six views in each condition were averaged and expressed as mean ± standard deviation (SD).

### Biochemical analyses

FLS cells were washed twice with cold PBS in a culture medium, peeled off with a cell scraper under cold PBS, and collected. The recovered FLS cells were centrifuged, and the supernatant was removed and frozen. Subsequently, 100 μL of a cold radioimmunoprecipitation analysis buffer was added to the frozen cell pellets, which were suspended with a vortex mixer. Next, the cells were centrifuged (16,000 × *g*) for 10 min at 20 °C, and the middle layer was used as the protein extract. The proteins were quantified using a Takara BCA Protein Assay Kit (Cat. No: T9300A), with bovine serum albumin representing the standard, according to the manufacturer’s protocol. Each sample was assayed at a 20 × dilution. Loading samples were prepared by mixing the protein extracts with a 4 × Sample buffer (Thermo Scientific Cat. No: NP0007) and a 10 × Sample Reducing agent (Cat. No: NP0009). Dithiothreitol (DTT) was used as the reducing agent to cleave the S-S bonds in the protein samples. The loading samples were heat-treated for 5 min at 95 °C and cooled rapidly, and each sample was loaded onto a polyacrylamide gel. The polyacrylamide gel was a 5–20 % gel (ATTO E-T520L Cat. No: 2331830) operated at a constant voltage of 150 V for approximately 60 min. Blocking One (Nakalai; Cat. No: 03953–95) was used for blocking for 60 min at room temperature. The ORAI1 (1:2000 in 5 % Blocking One containing 0.1 % TBST, Takara Cat. No: T9141, Proteintech, 662223–1-Ig), STIM1 (1:5000 in 5 % Blocking One containing 0.1 % TBST; Takara Cat. No: T9141, Proteintech, 11565–1-AP), CRACR2A (1:5000 in 5 % Blocking One containing 0.1 % TBST; Takara Cat. No: T9141, Proteintech, 66787–1-Ig), and Anti-TRPV4 (1:5000 in 5 % Blocking One containing 0.1 % TBST, Takara Cat. No: T9141, Abcam, ab191580) antibodies containing 5 % Blocking One in 0.1 % TBST (Takara Cat. No: T9141) solution were incubated overnight at 4 °C.

The cells were washed four times with 0.1 % TBST (Takara Cat. No: T9141) for 5 min. The secondary antibodies used were anti-rabbit IgG and horseradish peroxidase (HRP)-conjugated (1:5000 in 5 % Blocking One containing 0.1 % TBST, Cell Signaling Technology Cat. No: 7074) and anti-mouse IgG and HRP-conjugated (1:5000 in 0.1 % TBST containing 5 % Blocking One; Cell Signaling Technology Cat. No: 7076S) antibodies for 1 h at room temperature, followed by incubation in 0.1 % TBST (Takara Cat. No: T 9141) for 5 min four times. The proteins were detected with C-DiGit (LI-COR 3600–00) using an Immobilon Western Millipore Cat. No: WBKLS0500. The images were quantified using the ImageJ software (NIH, Bethesda, MD, USA).

### Statistical analysis

All data were expressed as the mean ± SD. Statistical analyses were performed using an unpaired *t*-test and a two-way analysis of variance with a post-hoc Tukey test. Statistical significance was set at p < 0.05.

## Results

### [Ca^2+^]_i_ increased the responses of the FLS cells to SS

**Fig. 1:**
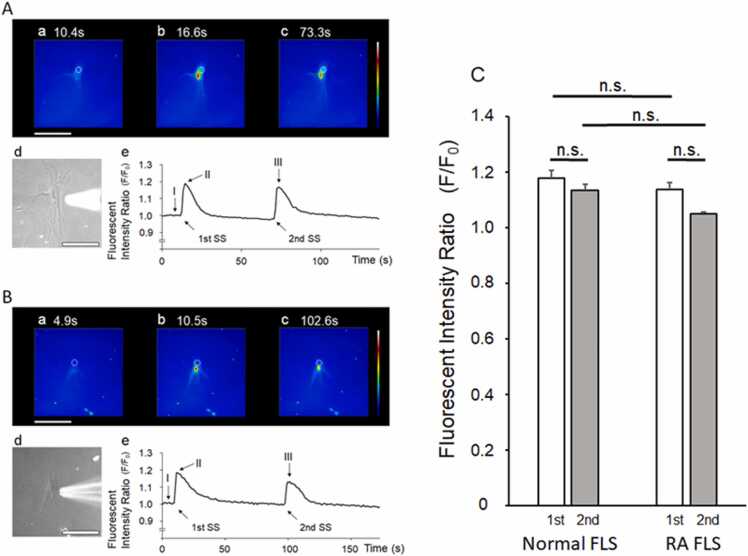
Typical [Ca^2+^]_i_ responses to two shear stress (SS) elicitations in human normal (A) and rheumatoid arthritis (RA; B) fibroblast-like synovial (FLS) cells Two [Ca^2+^]_i_ increasing responses were elicited by SS twice with puff applications. An immediate increase in the fluorescence intensity ratio was initiated at the stimulus site. The nucleus responded, and the response spread out of the cytosol around the nucleus (perikaryon). The peak fluorescence intensity ratios of the first and second responses were 1.19 and 1.17, respectively. The duration of the responses to the first and second SS were 36.1 and 42.4 s, respectively (A). The response of the RA FLS cells was spread from the nucleus to the cytosol, similar to that in the normal FLS cells. The peak fluorescence intensity ratios of the first and second responses were 1.18 and 1.13, respectively. The duration of the responses to the first and second SS were 56.5 and 37.7 s, respectively (B). a-c: Ca^2+^ fluorescence images. d: Phase-contrast image. e: Time-course changes in the fluorescence intensity ratio. Each fluorescence image (a-c) corresponds to the time points shown in I-III. I: pre-SS; II: peak ratio of the first SS; III: peak ratio of the second SS. Scale bar = 100 µm. The mean fluorescence intensity ratio of the first and second responses for the normal and RA FLS cells. The mean values of the fluorescence intensity ratios of the first and second responses for the normal FLS cells (n = 24) were 1.18 ± 0.15 and 1.14 ± 0.13, respectively. The mean values of the fluorescence intensity ratios of the first and second responses for the RA FLS cells (n = 23) were 1.13 ± 0.09 and 1.04 ± 0.03, respectively (C). n.s.: not significant.

This study aimed to investigate the response difference between normal and rheumatoid FLS cells. The [Ca^2+^]_i_ increase in the normal FLS cells was elicited by SS twice with puff application (Fig. 1A). An immediate increase in the fluorescence intensity ratio was observed at the stimulus site. This result suggests that the puff application via the micropipette was target-specific. The nucleus responded, and the response spread to the cytosol around the nucleus (perikaryon). The second response was lower than the first response. The response of the human RA FLS cells was similar to that of the normal FLS cells (Fig. 1B). The [Ca^2+^]_i_ increase began at the stimulus site and spread from the cytosol around the nucleus. The second response was lower than the first response; however, there were no significant differences in the response magnitudes (Fig. 1C).

### [Ca^2+^]_i_ increased the responses of the FLS cells to SS under Ca^2+^-free conditions

**Fig. 2:**
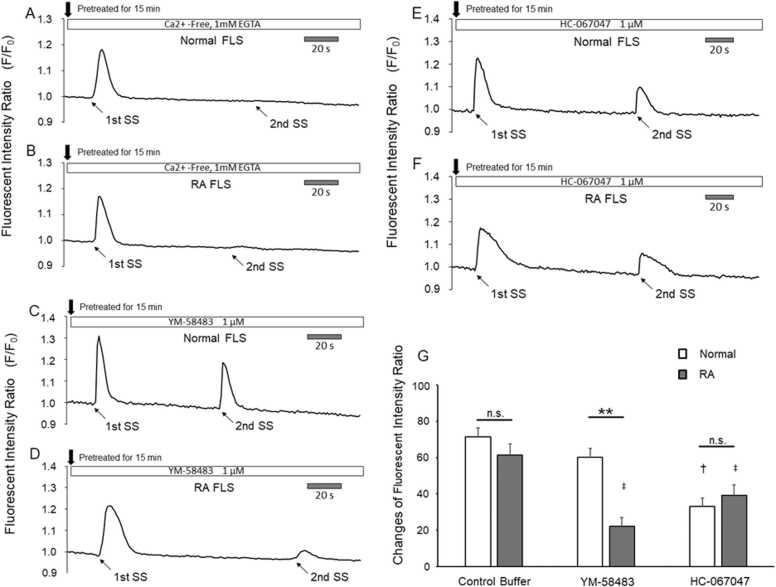
Responses to and changes in the second/first responses to two SS elicitations in normal and RA FLS, Time-course changes in the fluorescence intensity ratio of the normal (A) and RA (B) FLS cells under Ca^2+^-free conditions (superfused with 1 mM EGTA). The responses of the FLS cells to the first SS was sustained under Ca^2+^-free conditions. The responses to the second SS were abolished. The time-course changes in the fluorescence intensity ratio of the normal and RA FLS cells treated with YM-58483 (1 μM). The second response of the normal FLS cells was slightly reduced compared with the first response (C). In contrast, the second response of the RA FLS cells was markedly decreased compared with the first response (D). The time course changes in the fluorescence intensity ratio of the normal (E) and RA (F) FLS cells. In the normal and RA FLS cells, two responses were observed under two SS elicitations. The second response in the RA FLS cells had the same intensity as that in the normal FLS cells. Superfusion with the control buffer decreased the second/first response ratio of the normal FLS cells (n = 24) to 71.4 ± 5.0 % and that of the RA FLS cells (n = 23) to 61.3 ± 6.2 %. Superfusion with the YM-58483 buffer decreased the second/first response ratio of the normal FLS cells (n = 35) to 60.1 ± 4.8 % and that of the RA FLS cells (n = 18) to 22.1 ± 4.9 %. Superfusion with the HC-067047 buffer decreased the second/first response ratio of the normal FLS cells (n = 35) to 33.1 ± 4.7 % and that of the RA FLS cells (n = 26) to 39.1 ± 5.8 % (G). n.s.: not significant * *: p < 0.01, compared between the normal and RA FLS cells under YM-58483 buffer perfusion, ^†^: p < 0.05 compared with the normal FLS cells in the control buffer perfusion, ^‡^: p < 0.05 compared with the RA FLS cells in the control buffer perfusion.

Responses to two SS applications in the normal (Fig. 2A) and RA (Fig. 2B) FLS cells under extracellular Ca^2+^-free conditions were observed, as well as the time-course changes in the fluorescence intensity ratio of both FLS cell types under Ca^2+^-free conditions superfused with 1 mM EGTA. The first SS responses of the FLS cells were sustained under Ca^2+^-free conditions; however, the second SS responses were abolished.

### Effects of YM-58483 or HC-067047 on the FLS cell responses

Under two SS applications and YM-58483 perfusion (1 μM), the second response of the normal FLS cells was decreased compared with the first response, similar to the second response under the control buffer. This indicates that YM-58483 did not alter the second response in the normal FLS cells (Fig. 2C). In contrast, the second response of the RA FLS cells to YM-58483 was markedly decreased compared with the first response (Fig. 2D). When SS was applied twice and HC-067047 (1 μM) superfusion was performed, the first and second response patterns of the normal and RA FLS cells were similar to those of the control buffer (Fig. 2E). The extent of the second response of the RA FLS cells was similar to that of the second response of the normal FLS cells (Fig. 2F).

### Fluorescence intensity ratio of the first and second responses of FLS cells in control and Ca^2+^-Free buffers

There were no significant differences between the first (1.18 ± 0.15) and second (1.13 ± 0.13) responses in the normal FLS cells in the control buffer (n = 24). The second response (1.04 ± 0.03) in the RA FLS cells (n = 23) was significantly lower than the first response (1.13 ± 0.09) (p < 0.01). In the Ca^2+^-free buffer, the second response (1.00 ± 0.01E^−1^) in the normal FLS cells (n = 8) was significantly decreased compared with the first response (1.15 ± 0.05) (p < 0.05). The second response (1.00 ± 0.09E^−2^) in the RA FLS cells (n = 8) was significantly lower than the first response (1.12 ± 0.05) (p < 0.05).

### Fluorescence intensity ratio of the first and second responses of FLS cells superfused with a CRACI or TRPV4 blocker buffer

There were no significant differences between the first (1.16 ± 0.10) and second (1.09 ± 0.07) responses in the normal FLS cells (n = 35) under YM-58483 buffer superfusion; however, the second response (1.02 ± 0.02) was significantly lower than the first response (1.12 ± 0.05) in the RA FLS cells (n = 18) (p < 0.01). Under HC-067047 buffer superfusion, the first (1.21 ± 0.13) and second (1.07 ± 0.09) responses in the normal FLS cells (n = 35) showed no significant differences; however, the second response (1.05 ± 0.05) (n = 26) was significantly lower than the first response (1.19 ± 0.14) in the RA FLS cells (p < 0.01).

### Changes in the second/first response to two SS applications in the normal and RA FLS cells under superfusion with control, YM-58483, or HC-067047 containing a HEPES buffer

Changes (%) in the fluorescence intensity ratio of the second to the first responses among the control buffer, YM-58483, and HC-067047 groups were compared between the RA and normal FLS cells (Fig. 2G). In the control buffer treatment, the second responses, relative to the first responses, were decreased to 71.4 ± 5.0 % and 61.3 ± 6.2 % in the normal (n = 24) and RA (n = 23) FLS cells, respectively. There were no significant differences between normal and RA FLS cell responses. Superfusion with YM-58483 decreased the second/first response ratio in the normal FLS cells (n = 35) to 60.1 ± 4.8 % and significantly decreased (p < 0.01) those of the RA FLS cells (n = 18) to 22.1 ± 4.9 %. This indicates that YM-58483 markedly decreased the second/first response ratio in the RA FLS cells, suggesting that CRAC activation influenced the response of the RA FLS cells.

Superfusion with HC-067047 decreased the second/first response ratio of the normal FLS cells (n = 35) to 33.1 ± 4.7 % and decreased that of RA FLS cells (n = 26) to 39.1 ± 5.8 %. There were no significant differences in the responses between the normal and RA FLS groups. In contrast, the responses of normal and RA FLS cells were significantly smaller (p < 0.05) than those under the control buffer conditions. This suggests that TRPV4 activation exerted the same influence on the responses of the normal and RA FLS cells.

### Immunocytochemical analysis using anti-CRACR2A and anti-TRPV4 antibodies

**Fig. 3:**
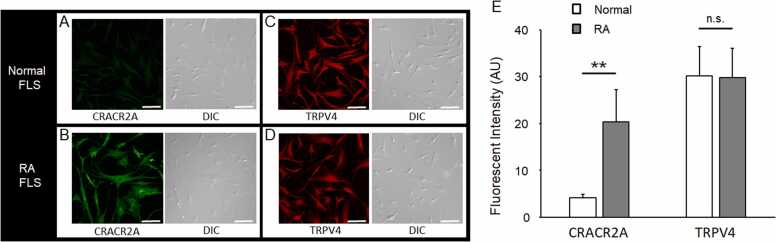
Immunofluorescence staining, differential interference contrast (DIC) microscopy images, and immunofluorescence intensity of the normal and RA FLS cells, Immunofluorescence staining with anti-Ca^2+^ release-activated channel regulator 2 A (CRACR2A) antibody (green, Alexa) and DIC images of the normal (A) and RA (B) FLS cells. Immunofluorescence staining with the anti-transient receptor potential vanilloid 4 (TRPV4) antibody (red, Alexa) and DIC images of the normal (C) and RA (D) FLS cells. All fluorescence and DIC images were obtained at 20 × magnification using an Olympus FV1000 Confocal Laser Microscope. Scale bar = 100 µm. The mean immunofluorescence intensity of the anti-CRACR2A antibody staining was significantly lower in the normal FLS cells (4.08 ± 0.8 AU) than in the RA FLS cells (20.3 ± 6.9 AU) (p < 0.01). The mean immunofluorescence intensities produced by the anti-TRPV4 antibody staining were not significantly different between the normal (30.3 ± 6.2 AU) and RA (29.7 ± 6.2 AU) FLS cells (E). n.s.: not significant, * *: p < 0.01^,^ compared between the normal and RA FLS cells.

Representative immunofluorescence staining and differential interference contrast microscopy images of the normal and RA FLS cells are shown in Fig. 3A–D. The mean immunofluorescence intensity of anti-CRACR2A antibody-positive cells was significantly lower in the normal FLS cells (4.08 ± 0.8 AU) than in the RA FLS cells (20.3 ± 6.9 AU) (p < 0.01). The mean immunofluorescence intensities of anti-TRPV4 antibody staining were not significantly different between the normal (30.3 ± 6.2 AU) and RA (29.7 ± 6.2 AU) FLS cells (Fig. 3E).

### Expression levels of ORAI1, STIM1, CRACR2A, and TRPV4 proteins

**Fig. 4:**
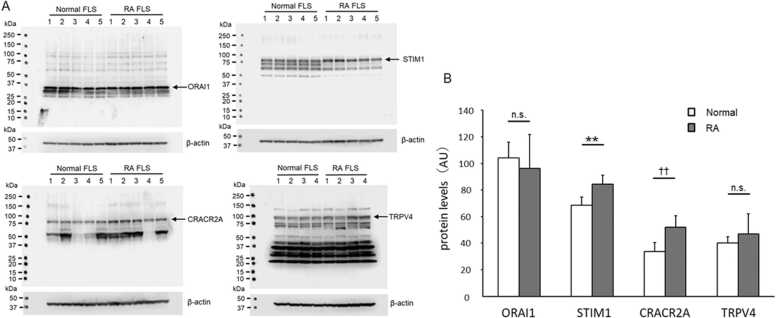
Expression levels of ORAI1, stromal interaction molecule 1 (STIM1), CRACR2A, and TRPV4 in the normal and RA FLS cells Western blotting of ORAI1, STIM1, CRACR2A, and TRPV4 expression in the normal and RA FLS cells. (A) The internal controls and representative western blots are shown. Subsequently, 10 or 30 μg of protein per lane was loaded onto the gel. Next, the membranes were probed with specific antibodies against Orai1, STIM1, CRACR2A, or TRPV4 with appropriate secondary antibodies. Protein bands were detected using C-DiGit (LI-COR#3600–00), and the results were analyzed using the ImageJ software (B). The protein amounts were normalized to β-actin amounts to indicate the protein expression ratios. * * and † † represent significant differences from normal FLS, respectively, at p < 0.01.

The protein levels of ORAI1, STIM1, CRACR2A, and TRPV4 were determined via western blotting (n = 5 for the normal and RA FLS cells for ORAI1, STIM1, and CRACR2A; n = 4 for TRPV4). The representative blots are shown in Fig. 4A. The expression levels of ORAI1, STIM1, and CRACR2A in the normal and RA FLS cells were 104.07 ± 12.08 and 96.30 ± 25.31, 68.46 ± 6.09 and 84.48 ± 6.72, and 38.81 ± 6.77 and 52.04 ± 8.55 AU, respectively, indicating that the proteins, except for ORAI1, exhibited significantly higher expression in the RA FLS cells than in the normal FLS cells (p > 0.01) (Fig. 4B). The TRPV4 levels in the normal (40.14 ± 4.73 AU) and RA (46.92 ± 15.01 AU) FLS cells were not significantly different.

## Discussion

To the best of our knowledge, this is the first report to indicate that the functional activation of CRAC is more dominant in RA FLS cells than in normal FLS cells. This study showed that STIM1 and CRACR2A expression levels were significantly higher in patients with RA than in those with normal FLS. The results of CRAC blockade with YM-58483 suggest the significance of CRAC in calcium influx in RA FLS cells. Fluid flow-induced mechanical stress has been used to stimulate cells in previous studies [8], [9], [10]. However, fluid flow stimulation is not cell-specific, affecting multiple cells. We developed an experimental system that applies SS to a single cell by expelling nitrogen gas. The first increase in the intracellular Ca^2+^ concentration in the Ca^2+^ imaging was related to the release of Ca^2+^ from the ER. This is because under extracellular Ca^2+^-free buffer conditions, although the first response was observed, there was no second response. In the first response, the Ca^2+^ stored in the ER was released; however, the Ca^2+^ (originally stored) concentration in the ER remains unknown. We could not determine whether the response that increased the intracellular calcium concentration was owing to calcium ion release from the ER or the influx from the outside of the cell. Therefore, the second/first response ratio was calculated to clarify the effects of administering the CRAC inhibitor and TRPV4 blocker [19], [25].

In this study, in the RA FLS cells, the second/first response ratio under perfusion with YM-58483 was significantly decreased compared with that under the control buffer treatment. We considered that the second response was significantly decreased by the CRAC inhibitor; however, it is unclear whether the Ca^2+^ in the ER was depleted during the first or second response. In addition, the expression levels of STIM1 and CRACR2A were significantly higher in the RA FLS cells than in the normal FLS cells. Orai1 and STIM1 form the STIM1-Orai1 complex when the Ca^2+^ in the ER is depleted, resulting in Ca^2+^ influx into the cells [26], [27]. CRACR2A is an intracellular Ca^2+^ sensor that modulates the STIM1-Orai1 complex and regulates CRAC channel activity, and the association of the CRACR2A-STIM1-Orai1 complex functions in a [Ca^2+^]i-dependent manner in the ER. CRACR2A dissociates from Orai1 and STIM1 and inhibits SOCE when [Ca^2+^]i is high, while low [Ca^2+^]i enhances CRACR2A binding to STIM1 and Orai1 and promotes SOCE [28].

As reported in previous studies on the therapeutic approaches for such inflammation, YM-58483 treatment or Orai1 and Orai3 knockout and the resultant decrease in Ca^2+^ influx suppressed inflammation in the synovial cells of collagen-induced arthritis mice [29], [30]. Previous studies have shown that CRACR2A overexpression enhances SOCE in T and HeLa cells overexpressing STIM1 and Orai1 [29]. These results suggest that the regulation of intracellular Ca^2+^ concentrations in RA FLS cells is CRAC-dependent. Several therapeutic approaches have targeted T cells for RA treatment [29]. In the synovial joints in RA, T and FLS cells communicate, and the cell contact between T cells and synovial fibroblasts induces cytokine release [31], [32]. CRAC inhibitors may influence the pathogenesis of RA by reducing the response of synoviocytes and their over-communication with T cells. This suggests that CRAC inhibitors may be effective for treating RA.

The TRPV family is activated by nitric oxide, elevated temperatures, pH changes, mechanical stimulation, and osmotic pressure. TRPV4 in the TRPV family participates in the intracellular Ca^2+^ dynamics induced by mechanical stimulations, and TRPV4 is an osmotic pressure or a mechanical sensor in vivo [33], [34], [35]. The intracellular Ca^2+^ concentration upon the mechanical stimulation of cells is regulated by the Ca^2+^ released from the ER via IP3 receptors and by Ca^2+^ signaling mechanisms through physical and functional interactions between TRPV4 and IP3 receptors [36].

**Fig. 5:**
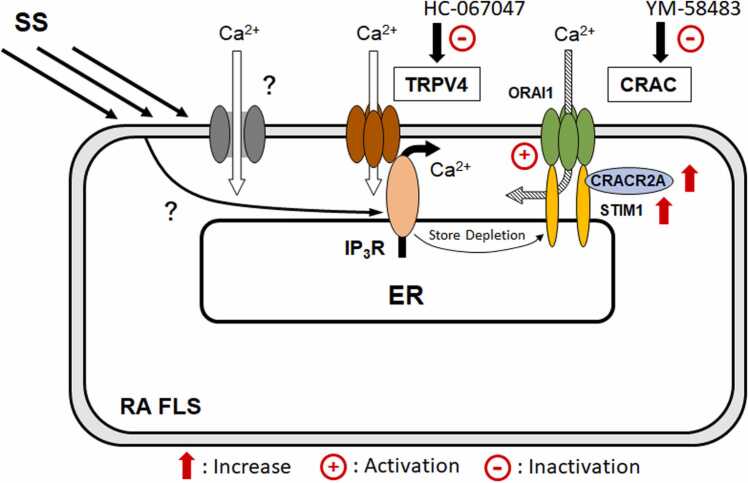
Ca^2+^ influx from the CRAC and TRPV4 of synovial cells in response to SS, When the Ca^2+^ in the endoplasmic reticulum was depleted by SS, CRACR2A directly interacted with the cytoplasmic regions of ORAI1 and STIM1 to form a ternary complex, resulting in the intracellular Ca^2+^ uptake from extracellular sources. In the RA FLS cells, STIM1 and CRACR2A were overexpressed compared with the expression in the normal FLS cells, and the regulation of intracellular Ca^2+^ concentration significantly depended on CRAC. SS also induced Ca^2+^ uptake from the extracellular into the intracellular site via TRPV4 and/or other mechanosensitive Ca^2+^ channels. However, there were no significant difference in the expression level of TRPV4 between the normal and RA FLS cells, indicating that TRPV4 exerted the same influence on the regulation of intracellular Ca^2+^ concentration in both FLS cell types.

In this study, we investigated the function and expression of TRPV4 to quantify its influence on the regulation of intracellular Ca^2+^ concentration under SS. HC-067047 superfusion caused no significant differences in the second/first response ratio or TRPV4 expression between the normal and RA FLS cells. These results suggest the same level of Ca^2+^ influx into cells via TRPV4 in the normal and RA FLS cell responses to SS (Fig. 5).

This study implicates CRAC-induced Ca^2+^ influx into synovial cells in the pathogenesis of RA. Further research on the influence of CRAC inhibitors on synovial cells may help explain their therapeutic effects on RA in synovial tissues. The SS-induced response was suppressed by GsMTx-4, a mechanosensitive ion channel inhibitor (data not shown). However, the interpretation of the results needs to be taken into account, as it differs from actual in vivo mechanical stress. In RA pathology, synovial cells secrete the inflammatory cytokines IL-6 and IL-8 [37]. The IL-6 concentration in the cell culture medium was not measured in this study. Therefore, the reactivity under IL-6 conditions remains unverified. Thus, the functions of other synovial cell channels on SS should be examined in future studies.

## Conclusions

This study showed that STIM1 and CRACR2A expression levels were significantly higher in patients with RA than in those with normal FLS. Our findings suggest that the activation of CRAC and Ca^2+^ influx in RA FLS cells participate in the pathogenesis of RA.

## Abbreviations

CRAC, Calcium release-activated calcium channel; CRACR2A, Ca^2+^ release activated channel regulator 2A; DMARDs, Disease-modifying antirheumatic drugs; DMEM, Dulbecco’s modified Eagle’s medium; DTT, Dithiothreitol; FLS, Fibroblast-like synovial cell; GM-CSF, Granulocyte-macrophage colony-stimulating factor; IL-1β, Interleukin-1 beta; IL-6, Interleukin-6; IL-8, Interleukin-8; Orai1, Calcium release-activated calcium modulator 1; RA, Rheumatoid arthritis; RANKL, Receptor activator of nuclear factor-kappa B ligand; SS, Shear stress; STIM1, Stromal interaction molecule 1; SOCE, Store-operated Ca^2+^ entry; TRPV4, Transient receptor potential vanilloid 4.

## Funding

This work was supported by JSPS KAKENHI Grant Numbers JP19K11335, JP20K11238, JP20K11297.

## CRediT authorship contribution statement

**Okumura Yu:** Writing – review & editing, Writing – original draft, Visualization, Validation, Methodology, Investigation, Funding acquisition, Formal analysis, Data curation, Conceptualization. **Honoki Kanya:** Writing – review & editing, Writing – original draft, Supervision, Software, Resources, Project administration. **Tanaka Yasuhito:** Writing – review & editing, Writing – original draft, Supervision, Software, Resources, Project administration. **Takaki Miyako:** Writing – review & editing, Writing – original draft, Visualization, Validation, Supervision, Software, Resources, Project administration, Methodology, Investigation, Funding acquisition, Formal analysis, Data curation, Conceptualization. **Asada Keiji:** Writing – review & editing, Writing – original draft, Visualization, Validation, Supervision, Project administration, Methodology, Investigation, Funding acquisition, Formal analysis, Data curation, Conceptualization.

## Declaration of Competing Interest

The authors declare that they have no known competing financial interests or personal relationships that could have appeared to influence the work reported in this paper.

## Data Availability

The data that support the findings of this study are available from the corresponding author, upon reasonable request.
